# Future risk of cardiovascular disease risk factors and events in women after a hypertensive disorder of pregnancy

**DOI:** 10.1136/heartjnl-2018-313453

**Published:** 2019-06-07

**Authors:** Laura Benschop, Johannes J Duvekot, Jeanine E Roeters van Lennep

**Affiliations:** 1 Department of Obstetrics and Gynaecology, Erasmus MC, Rotterdam, The Netherlands; 2 Department of Epidemiology, Erasmus Medical Center, Rotterdam, The Netherlands; 3 Department of General Medicine, Erasmus Medical Center, Rotterdam, The Netherlands

**Keywords:** cardiac risk factors and prevention, pregnancy, hypertension, lipoproteins and hyperlipidemia, diabetes

## Abstract

Hypertensive disorders of pregnancy (HDP), such as gestational hypertension and pre-eclampsia, affect up to 10% of all pregnancies. These women have on average a twofold higher risk to develop cardiovascular disease (CVD) later in life as compared with women with normotensive pregnancies. This increased risk might result from an underlying predisposition to CVD, HDP itself or a combination of both. After pregnancy women with HDP show an increased risk of classical cardiovascular risk factors including chronic hypertension, renal dysfunction, dyslipidemia, diabetes and subclinical atherosclerosis. The prevalence and onset of cardiovascular risk factors depends on the severity of the HDP and the coexistence of other pregnancy complications. At present, guidelines addressing postpartum cardiovascular risk assessment for women with HDP show a wide variation in their recommendations. This makes cardiovascular follow-up of women with a previous HDP confusing and non-coherent. Some guidelines advise to initiate cardiovascular follow-up (blood pressure, weight and lifestyle assessment) 6–8 weeks after pregnancy, whereas others recommend to start 6–12 months after pregnancy. Concurrent blood pressure monitoring, lipid and glucose assessment is recommended to be repeated annually to every 5 years until the age of 50 years when women will qualify for cardiovascular risk assessment according to all international cardiovascular prevention guidelines.

## Introduction

Cardiovascular disease (CVD) is currently the main cause of death among men and women worldwide.^1^ Although certain cardiovascular risk factors are already apparent at a younger age, women develop CVD on average 10–15 years later than men.^1^ This provides a window of opportunity to treat cardiovascular risk factors at a younger age and reduce the risk of developing CVD. One of these early cardiovascular risk factors is a hypertensive disorder of pregnancy (HDP), such as gestational hypertension and pre-eclampsia. In this review, we will summarise available data on the development of and follow-up for CVD risk factors and events among women with a history of HDP. We will also provide a practical approach to cardiovascular follow-up after pregnancy for these women.

### Hypertensive disorder of pregnancy: definition and epidemiology

Table 1 gives an overview of the different types of hypertension that may occur during pregnancy. Chronic hypertension, gestational hypertension and (superimposed) pre-eclampsia or eclampsia are referred to as HDP.^2^ This review will focus on HDP conditions involving new-onset high blood pressure in pregnancy: gestational hypertension and pre-eclampsia. The prevalence increased by 25% in the last decade and is expected to continue to grow with the rise of risk factors associated with developing HDP, such as diabetes, obesity and advanced maternal age. This is expected to also increase the rates of adverse obstetric outcomes associated with HDP, including fetal growth restriction, preterm birth and perinatal death.^3^ In the USA, HDP account for 7.4% of maternal deaths due to the increased risk of renal failure, pulmonary oedema, respiratory distress syndrome and stroke.^4–6^ These women are also more at risk to develop CVD later in life.^7^ Although the exact underlying pathophysiology for this excessive CVD risk remains unknown, the current literature suggests three pathways: 1) pregnancy-induced CVD risk, 2) a prepregnancy predisposition towards an increased risk of CVD or 3) a combination of both pathways.^8^

**Table 1:** Types of hypertension that can occur in pregnancy^2^

Hypertension	Definition	Prevalence	Risk factors
Chronic hypertension	SBP≥140 or DBP≥90 mm Hg before pregnancy or before 20 weeks of gestation	14% of pregnancies	Obesity, a family history of hypertension, advanced maternal age.
Gestational hypertension	SBP≥140 or DBP≥90 mm Hg after 20 weeks of gestation	2%–5% of pregnancies	(Pre)gestational diabetes, pre-eclampsia in a previous pregnancy, nulliparity, twin pregnancy, obesity, pregnancy via assisted reproductive technology and born of an HDP pregnancy
Pre-eclampsia	SBP≥140 or DBP≥90 mm Hg after 20 weeks of gestation and the presence of proteinuria (≥300 mg/day or ≥1 g/L on dipstick testing), maternal organ dysfunction (renal insufficiency, liver involvement, neurological complications (including eclampsia) or thrombocytopenia) or fetal growth restriction	2%–5% of pregnancies	Those mentioned under gestational hypertension and antiphospholipid antibody syndrome, maternal age<18 or >35 years, black race, first degree relative with pre-eclampsia, migraine, SSRI use after the first trimester, thrombophilia, chronic kidney disease and autoimmune disease
Superimposed pre-eclampsia	SBP≥140 or DBP≥90 mmHg before pregnancy or before 20 weeks of gestation with a new-onset proteinuria or an acute exacerbation of hypertension or proteinuria in the second half of pregnancy or sudden systemic features of pre-eclampsia		Previous pre-eclampsia

DBP, diastolic blood pressure; HDP, hypertensive disorder of pregnancy; SBP, systolic blood pressure; SSRI, selective serotonin reuptake inhibitor.

## Cardiovascular risk factors after pregnancy

### Chronic hypertension

Compared with women with normotensive pregnancies, women with HDP have a twofold to eightfold increased risk of future chronic hypertension.^9–18^ This was confirmed by a recent Danish register-based cohort study which included 1.5 million primiparous women with information on HDP and chronic hypertension diagnosis during a follow-up time of 1–20 years.^9^ Women with gestational hypertension had the highest risk of developing chronic hypertension after pregnancy, followed by women with severe pre-eclampsia and moderate pre-eclampsia. Taking into account confounding factors the risk of chronic hypertension was 4–10 times higher in women with an HDP compared with women with a normotensive pregnancy 1–5 years after pregnancy. By the age of 50 years, this risk became similar between the different HDP; a 2-fold to 2.5-fold higher risk compared with women with a previous normotensive pregnancy. Up to 32% of women with HDP developed chronic hypertension in the first 10 years after pregnancy versus up to 11% of women with normotensive pregnancies, depending on their age during the first pregnancy. Eight years post partum, 10% of women aged 20–29 years with a previous HDP had chronic hypertension, which is comparable to the prevalence of chronic hypertension of women with previous normotensive pregnancies aged 40–49 years 10 years post partum. A meta-analysis by Heida *et al* demonstrated that women with pre-eclampsia have a relative risk (RR) 2.76 (95% CI 1.63 to 4.69) increased risk of having chronic hypertension after pregnancy compared with women with a normotensive pregnancy.^10^ This risk was comparable for women with gestational hypertension (RR 2.87, 95% CI 0.84 to 9.77). These results are in line with other large studies which show that the relative risk of having chronic hypertension is especially high shortly after pregnancy and eventually plateaus.^11^

Several studies also examined the severity of the HDP in relation to the risk of developing chronic hypertension after pregnancy.^9 13–15^ study by Behrens *et al* demonstrated that women with severe pre-eclampsia had a higher risk to develop chronic hypertension 1 year after pregnancy (HR 6.45, 95% CI 5.35 to 7.78) than women with moderate pre-eclampsia (HR 5.25, 95% CI 4.64 to 5.94).^9^ We recently showed that 1 year post partum, 42.5% of women with severe pre-eclampsia, assessed by ambulatory blood pressure monitoring, had night-time hypertension and 44.5% had an insufficient decrease in systolic blood pressure at night-time compared with daytime. Both conditions are associated with an increased risk of CVD.^16 17^

The risk of chronic hypertension also depends on the number of pregnancies affected by HDP as was recently demonstrated in a meta-analysis by Brouwers *et al*.^18^ Women with recurrent pre-eclampsia had a higher risk of chronic hypertension after pregnancy than women with a subsequent uncomplicated pregnancy after a pre-eclamptic pregnancy (RR 2.3, 95% CI 1.9 to 2.9).

### Renal dysfunction

Microalbuminuria is a persistent, increased urinary excretion of albumin and is recognised as a marker for renal dysfunction and a risk factor for CVD.^19 20^ A meta-analysis of 606 women showed that 7.1 (95% CI 4.5 to 9.7) years post  partum those with a history of pre-eclampsia, taking into account chronic hypertension and diabetes, have a fourfold increased risk of microalbuminuria compared with women with uncomplicated pregnancies (31% vs 7%, respectively).^21^ Both in and outside pregnancy, a negative association between blood pressure levels and renal function was observed. This might explain why especially women with pre-eclampsia are at risk for impaired renal function.^22 23^ A recent Canadian population-based follow-up study examined the risk of end-stage renal disease (ESRD) among 1.5 million women over a median follow-up time of 16.2(IQR 13.3–18.3) years.^22^ This study showed that the absolute risk of ESRD is very low; 0.15% for women with a previous HDP vs 0.03% for women with a previous normotensive pregnancy.^22^ After partial adjustment for age and region, women with a history of pre-eclampsia were most at risk of ESRD after pregnancy (HR 4.7, 95% CI 3.6 to 6.0), followed by women with a history of gestational hypertension (HR 3.3, 95% CI 2.1 to 5.1) compared with women with previous normotensive pregnancies. The risk of ESRD increases when multiple pregnancies are affected by pre-eclampsia, as was previously demonstrated in a large register-based Norwegian study which adjusted for results for main confounders including age and year of delivery.^24^ Women with pre-eclampsia in their first pregnancy had a RR for ESRD of 4.7 (95% CI 3.6 to 6.1), whereas women with pre-eclampsia in more than one pregnancy had a RR of 15.5 (95% CI 7.8 to 30.8).^24^

### Dyslipidemia

Women with HDP are at increased risk for having an adverse lipid profile after pregnancy than women with a normotensive pregnancy.^13 25 26^ A meta-analysis of 15 studies, including 736 women with a previous HDP and 701 women with previous normotensive pregnancies showed that the former have more often dyslipidemia than women with normotensive pregnancies (pooled unadjusted mean differences varied between 0.13 and 0.22 mmol/L).^25^ We recently showed that dyslipidemia was more frequent in women with a history of HDP than in women with normotensive pregnancies 6 years after delivery in a group of 4933 women.^26^ After adjustment for all relevant confounders, total-cholesterol, triglycerides, high-density lipoprotein-cholesterol, low-density lipoprotein-cholesterol, lipoprotein(a) and apolipoprotein B levels were all higher in women with previous gestational hypertension compared with women with a previous normotensive pregnancy. Women with previous pre-eclampsia had higher triglyceride levels compared with women with a previous normotensive pregnancy. Nevertheless, this risk was predominantly driven by prepregnancy body mass index (BMI).

### Diabetes

In addition to an increased risk of insulin resistance during pregnancy, women with HDP are also more at risk of developing diabetes later in life. A large Canadian study retrospectively examined the risk of diabetes after pregnancy partially adjusted for relevant confoundersand comorbidity, in 1 million women with a median follow-up of 8.5 years.^27^ Women without gestational diabetes, were more likely to develop diabetes after gestational hypertension (3.9%) or pre-eclampsia (6.6%)than after a normotensive pregnancy (2.5%). A register-based cohort study from Denmark also demonstrated that the fully adjusted risk of type 2 diabetes 14.6 years after pregnancy (range 0.25–30.2) was 3.12-fold (range 2.63–3.70) for women with gestational hypertension and 3.68 (range 3.04–4.46) after pre-eclampsia.^15^

### Subclinical atherosclerosis

Both during and after pregnancy, women with pre-eclampsia have a larger carotid intima media thickness (CIMT) than women without pre-eclampsia.^28 29^ Several studies have shown that the coronary artery calcium score (CACS) is higher in women who had previous pre-eclampsia compared with women without a hypertensive pregnancy.^30 31^ Most of these studies were small and performed in women of approximately 60 years, >30 years after pregnancy. In the Cardiovascular Risk Profile: Imaging and Gender-Specific Disorders (CREW-IMAGO)study, we showed that 31% of women with previous pre-eclampsia aged 45–55 years had a CACS of >0 in comparison with 18% of women with a previous normotensive pregnancy who were matched for gender, age and ethnicity.^26^ Moreover, 47% of women with previous pre-eclampsia had coronary atherosclerotic plaques on coronary computed tomography angiography (CCTA) and 4.3% significant stenosis.^26^

### Cardiovascular disease

Multiple meta-analyses based on cohort studies showed that women with HDP have an increased risk of CVD that was not explained by adjustment for confounding variables.^7 10 21 32 33^ In women with previous pre-eclampsia, the RR of coronary heart disease ranged between 2.06 and 2.50^7 10 21 32 33^ and the RR of cerebrovascular disease between 1.53 and 3.13.^7 10 21 33^ Peripheral artery disease was only assessed in one meta-analysis, finding a 1.87-fold increased risk (95% CI 0.94 to 3.37) among women with a history of pre-eclampsia or eclampsia based on three studies.^21^ The CVD risk also depends on specific pregnancy characteristics. For instance, women with early onset pre-eclampsia before 37 weeks of gestation have a markedly increased risk of CVD compared with women with late-onset pre-eclampsia (RR 7.71, 95% CI 4.40 to 13.52 vs RR 2.16, 95% CI 1.86 to 2.52).^32^ Moreover, women who had pre-eclampsia in combination with a child born small for gestational age or a preterm delivery have an increased risk of CVD compared with women with only pre-eclampsia after adjustment for confounders (HR 3.3, 95% CI 2.37 to 4.57 vs HR 5.38, 95% CI 3.74 to 7.74 and HR 2.14, 95% CI 1.73 to 2.65, respectively).^34^ After a pregnancy complicated by pre-eclampsia the risk of recurrent pre-eclampsia in a subsequent pregnancy is approximately 15%.^35^ Women with recurrent pre-eclampsia have an increased risk of ischaemic heart disease (RR 2.40, 95% CI 2.15 to 2.68), cerebrovascular disease (RR 1.69, 95% CI 1.21 to 2.35) and cardiovascular events and hospitalisation (RR 1.57, 95% CI 1.31 to 1.90) compared with women with a subsequent uncomplicated pregnancy.^18^ Less data are available regarding the risk of CVD in women with gestational hypertension as compared with women with pre-eclampsia. A recent review showed that the risk of fatal and non-fatal CVD as well as ischaemic heart disease and stroke was higher in women who experienced gestational hypertension than in women with an uncomplicated pregnancy (RR 1.89, 95% CI 1.31 to 2.72, RR 1.44, 95% CI 1.30 to 1.60 and RR 1.41, 95% CI 1.20 to 1.65, respectively).^10^ A large population-based study demonstrated that women with gestational hypertension had a 1.8-fold (95% CI 1.7 to 2.0) higher risk of developing subsequent CVD, a 1.7-fold (95% CI 1.3 to 2.1) higher risk of subsequent coronary heart disease and a 1.3-fold (95% CI 0.9 to 1.7) higher risk of developing cerebrovascular disease compared with women without an HDP.^36^ Furthermore, women with a gestational hypertension in combination with a child born small for gestational age and a preterm delivery had the highest CVD risk after adjustment for most confounders but not smoking, BMI and pre-existing hypertension (HR 2.3, 95% CI 2.3 to 3.0).^36^

### Approach to follow-up

**Figure 1:**
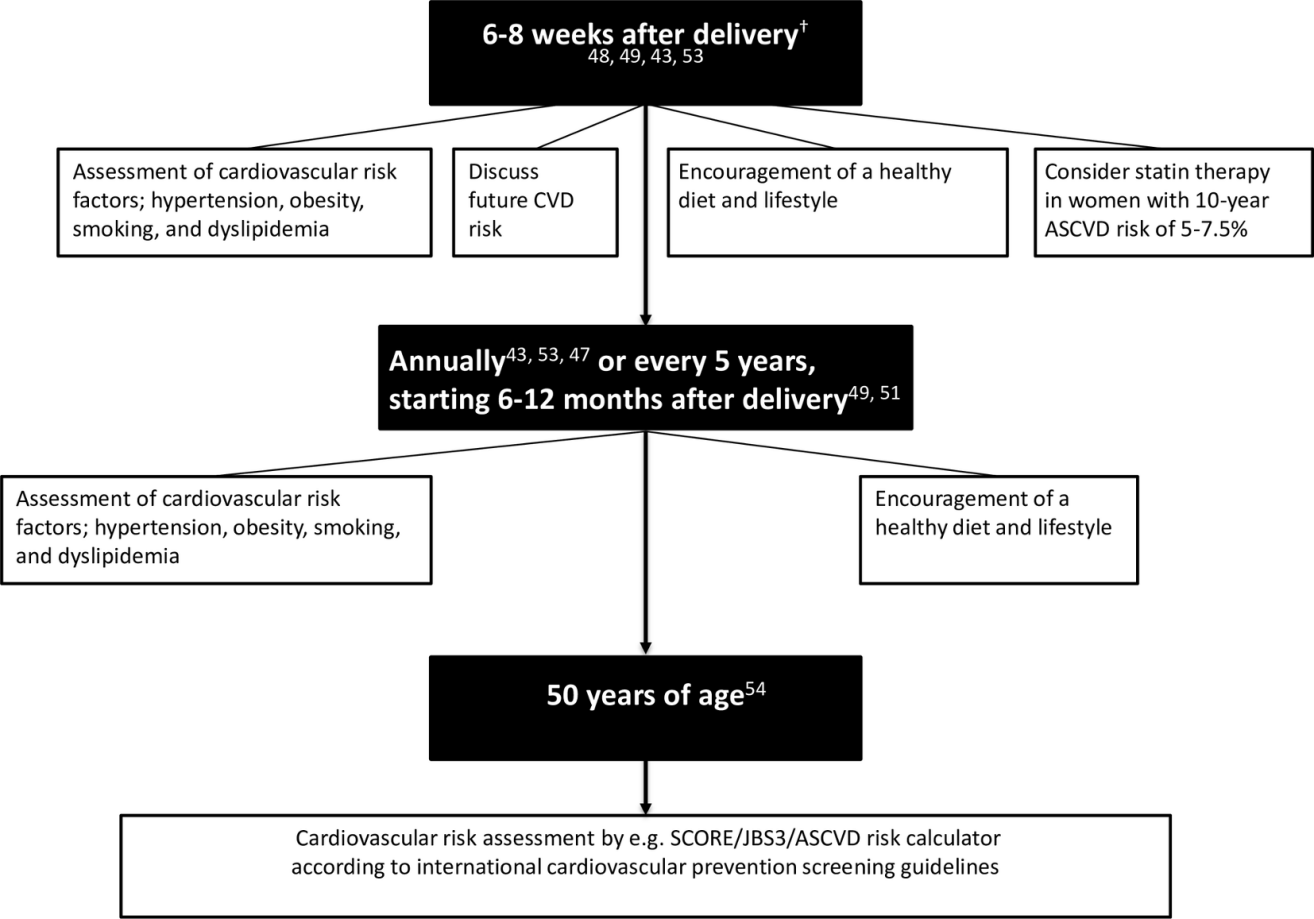
Schedule for suggested cardiovascular follow-up after a hypertensive disorder of pregnancy. ASCVD, atherosclerotic cardiovascular disease; BMI, body mass index; JBS3, Joint British Societies recommendations on the prevention of Cardiovascular Disease; SCORE, Systematic COronary Risk Evaluation. *National Institute for Health and Care Excellence. Hypertension in pregnancy, 2013 (updated 2017).

In summary, already 1 year after delivery women with HDP have relatively more cardiovascular risk factors than women after normotensive pregnancies. In general, women with pre-eclampsia, especially severe, early onset pre-eclampsia, have the most adverse cardiovascular risk phenotype. Also, studies show that women who had gestational hypertension have an increased cardiovascular risk profile. Women with a history of pre-eclampsia develop chronic hypertension and thereafter dyslipidemia earlier then women with previous normotensive pregnancies. By the age of 35 years, the number needed to screen (NNT) to diagnose one woman with chronic hypertension is 9 and the NNT to diagnose dyslipidemia by the age of 39 years is 18. For diabetes mellitus, the NNT is 22 between the age of 50 and 55 years.^37^ Subclinical atherosclerosis visible by cardiovascular imaging is already apparent by the age of 45 years, when women are on average 16.3±5.9 years post partum.^26^ This underlines that, to optimise cardiovascular prevention, it is important to initiate cardiovascular screening soon after delivery. Nevertheless, uniform recommendations regarding cardiovascular follow-up after HDP are lacking. This follow-up could be provided by various healthcare professionals, including general practitioners, internists, cardiologists or gynaecologists, all using different guidelines (table 2). Advise on how and when cardiovascular risk assessment should be carried out and by which healthcare provider is currently confusing as it differs between guidelines. At present, only half of the most commonly used guidelines advise cardiovascular risk assessment after pregnancy. Figure 1 provides an overview of these recommendations for cardiovascular follow-up after HDP.^9 14 26 27 38–43^ Initially, all women should be informed about their increased risk of future CVD after HDP.^44^ Healthcare providers should encourage a healthy diet and lifestyle, especially for overweight women.^45^ The American  Heart   Association and National Institute for Health and Care Excellence guidelines advise to perform a first cardiovascular follow-up 6–8 weeks post partum (table 2).^39 40^ As lipid levels increase during pregnancy and will gradually normalise, measuring lipids within 1 year post partum is not recommended. The American College of Obstetricians and Gynecologists, European Society of Cardiology/European Society of Hypertension and American Stroke Association guidelines advise cardiovascular follow-up 6–12 months post partum.^41–43^ Thereafter, some guidelines advise annual follow-up of blood pressure and metabolic factors by primary care physician,^42 46^ whereas others recommend to repeat follow-up every 5 years.^9 14 26 27 38 47^ At 50 years, all women including those with HDP will qualify for regular cardiovascular risk assessment according to all major international cardiovascular prevention guidelines.^26^ Besides obtaining a uniform approach to the cardiovascular follow-up of women with an HDP, it also remains a challenge to motivate women to attend a cardiovascular prevention programme. Evaluation of a cardiovascular screening programme in the UK observed that those adhering to the programme are mostly highly educated and often non-smokers.^48^ Although some cardiovascular risk factors have been identified, it remains difficult to predict who will develop CVD and who will not. Therefore, the development of clinical prediction models and biomarkers will help to personalise cardiovascular risk management in women after HDP.

**Table 2:** Cardiovascular follow-up after a hypertensive disorder of pregnancy

Guideline	Year	Follow-up CVD risk
Guidelines with no recommendations regarding cardiovascular follow-up		
WHO*	2011	None
ISSHP	-	None
Guidelines with no specific timeline recommendations regarding cardiovascular follow-up		
ACOG	2013 and 2018	Women with preterm delivery (<37 weeks) or recurrent pre-eclampsia: annual blood pressure, lipids, fasting glucose and BMI. No recommendation on starting time and which healthcare provider.
RCOG^44^	2006	Inform about increased CVD risk in the future.
SOGC^45^	2014	Assessment of traditional cardiovascular risk markers may be beneficial. Encourage a healthy diet and lifestyle, especially, for overweight women.
Guidelines with timeline recommendations regarding cardiovascular follow-up		
NICE†	2017	Discuss future CVD risk 6–8 weeks after pregnancy with healthcare provider.
ASA^43^	2014	Consider to evaluate and treat all women with a history of pre-eclampsia for cardiovascular risk factors such as hypertension, obesity, smoking and dyslipidemia, starting 6 months to 1 year post partum.
ESC/ESH^42 46^	2018	Annual check of blood pressure and metabolic factors by primary care physician.
SOMANZ^47^	2014	Cardiovascular risk assessment every 5 years.
AHA^39 49^	2011/2018	Postpartum referral by the obstetrician to a primary care physician or cardiologist to monitor and control cardiovascular risk factors. Consider statin therapy in women with 10-year ASCVD risk of 5%–7.5%.
NVOG^50^	2014	Cardiovascular risk assessment at the age of 50 years.

*WHO recommendations for prevention and treatment of pre-eclampsia and eclampsia, 2011.

†Hypertension in pregnancy, 2013 (updated 2017).

ACOG, American College of Obstetricians and Gynecologists; AHA, American Heart Association; ASA, American Stroke Association; ASCVD, atherosclerotic cardiovascular disease; ESC, European Society of Cardiology; ESH, European Society of Hypertension; ISSHP, International Society for the study of Hypertension in Pregnancy; NICE, National Institute for Health and Care Excellence; NVOG, Nederlandse Vereniging voor Obstetrie en Gynaecologie; RCOG, Royal College of Obstetricians and Gynaecologists; SOGC, Society of Obstetricians and Gynecologists of Canada; SOMANZ, Society of Obstetric Medicine Australia and New Zealand.

In conclusion, although women with HDP have more cardiovascular risk factors soon after pregnancy, the lag-time between pregnancy and the occurrence of CVD provides a unique window of opportunity for timely cardiovascular prevention. The current challenge is how to use this period most optimal. Therefore, it is time to focus research on optimising the implementation of cardiovascular follow-up programmes targeted to these women.
